# Demographic and Psychographic Factors of Social Isolation During the COVID-19 Pandemic: The Importance of Technology Confidence

**DOI:** 10.3389/fpubh.2021.749515

**Published:** 2021-10-28

**Authors:** Becky R. Horst, Andrew Sixsmith, Dorina Simeonov, Alex Mihailidis

**Affiliations:** ^1^Department of Neuroscience, Western University, London, ON, Canada; ^2^AGE-WELL NCE, Toronto, ON, Canada; ^3^STAR Institute, Department of Gerontology, Simon Fraser University, Burnaby, BC, Canada; ^4^Department of Occupational Science and Occupational Therapy, University of Toronto, Toronto, ON, Canada

**Keywords:** older adults, communication technology, confidence, demographic factors, social isolation, pandemic

## Abstract

The COVID-19 pandemic presents an unprecedented situation in which physical distancing and “stay at home” orders have increased the pressures for social isolation. Critically, certain demographic factors have been linked to increased feelings of isolation and loneliness. These at-risk groups for social isolation may be disproportionately affected by the changes and restrictions that have been implemented to prevent viral spread. In our analysis, we sought to evaluate if perceived feelings of social isolation, during the COVID-19 pandemic, was related to demographic and technology-related psychographic characteristics. Older adults across Canada were surveyed about their demographic background, their feelings concerning confidence and proficiency in technology use, and how frequently they have felt isolated during the pandemic. In total 927 responses from Canadians over 65 years old, of varying demographic characteristics were collected. Our data shows that many older adults are feeling isolated “Often” or “Some of the time” in 2020, regardless of most demographic factors that have been previously associated with increased isolation risk. However, feelings of proficiency in using technology was an important factor affecting feelings of isolation. Given that technology proficiency is a modifiable factor, and remained significant after adjustment for demographic factors, future efforts to reduce social isolation should consider training programs for older adults to improve technology confidence, especially in an increasingly digital world.

## Introduction

Feelings of social isolation and loneliness is a global public health concern that particularly affects older adults (1). It is well-established that many negative health consequences such as declines in cognitive function, mental health, decreased immune function, and mortality are associated with loneliness and social isolation in older adulthood (2–4). Related to this, a number of studies have begun to explore the sociodemographic characteristics that may predispose individuals to these feelings of isolation. Some of the demographic risk factors for loneliness and social isolation among older people include but are not limited to, disability, low income, living alone, poor health, less education, and certain racial or ethnicity differences (5, 6).

The COVID-19 pandemic presents an unprecedented situation in which physical distancing and “stay at home” orders have increased the pressures for social isolation. Many businesses, places of education, and recreational facilities were shuttered to prevent the spread of the virus, but also consequently cut off the potential for in-person social engagement through these outlets. As a result of these orders, we have also seen a large-scale transition to a heavy reliance on technology for social connectedness and everyday services (7, 8). With these changes, the impact on those who are demographically at risk for social isolation may be disproportionately amplified.

In our analysis, we sought to evaluate if perceived feelings of social isolation, during the COVID-19 pandemic, were related to demographic characteristics that have been previously considered to be more likely to experience social isolation. Furthermore, because of the current necessity of technology for social communication and services, we proposed to investigate the relationship between the psychographic characteristics of technology confidence and feelings of social isolation. Understanding the associations of sociodemographic and psychographic factors related to social isolation may aid in directing strategies and resources toward groups who are demonstrating heightened feelings of isolation during this pandemic.

## Materials and Methods

Participant recruitment and data collection were completed by Environics Research, a commercial company, commissioned by AGE-WELL NCE (www.agewell-nce.ca) in Canada. Data was provided to the authors for secondary analysis and approved by the Research Ethics Board at Simon Fraser University REB #30000195.

Canadian adults over the age of 50 were invited to complete the survey and answer questions on their attitudes and behaviors concerning technology. Sampling and recruitment were completed by the Environics research team. Specific quotas were outlined for each survey to ensure, (1) at least 45% of the responses collected were over the age of 65 years old; (2) 51% of the sample was female, and (3) the distribution of geographical responses aligned with proportional representation of the population. The surveys were offered in both French and English and responses were collected online, or via computer-assisted telephone interview (CATI). For our analysis, only the data of those aged 65 or older was processed.

Various self-reported demographic characteristics were collected to describe the sample: age; gender; living in a rural or urban setting; housing status (living alone or with others); relationship status (in a partnership or not); level of income (Low: $39,999 or less, Middle: $40,000–$99,999, or High: $100,000 or more); level of education (high school equivalent or less, technical degree or some post-secondary, completed university degree); self-identify as a Black, Indigenous or Person of Color (BIPOC); self-identify as a person with a disability.

Two psychographic measures were also collected to describe the sample. Survey participants were asked to rate on a Likert scale their confidence to use technology, as well as how “Tech-savvy” they felt there are. Responses for the technology confidence rating were sorted into “Confident,” those who reported some level of confidence, and “Not Confident,” those who reported some level of non-confidence. Similarly, those that responded that they felt some level of Tech-savviness were classified as “Tech-savvy” and those that reported negatively on the scale were classified as “Not Tech-savvy.”

To collect feelings of isolation, survey respondents were asked, “how often did you feel isolated from others?”. Response options were “Hardly ever,” “Some of the time,” or “Often.”

### Statistical Analysis

To examine the unadjusted bivariate relationship of demographic and psychographic characteristics on feelings of isolation we conducted chi-square testing (α = 0.05). Significant associations were further evaluated using Bonferroni corrected pairwise *z*-testing, where appropriate, to evaluate where proportions differed. We also performed unadjusted and adjusted multinomial logistic regression to test the overall effects and odds ratios of sociodemographic factors of feelings of isolation “hardly ever” and “some of the time” compared to those that reported social isolation “often.”

## Results

A total of 2,026 response were collected. For our analysis of responses aged 65 and over the sample included 927 total responses. Demographic and psychographic characteristics of the sample are reported in Table 1. Within the entire sample, 15.4% reported feeling isolated “Often,” 49.7% “Some of the time,” and 34.8% “Hardly ever” in the past few months, during COVID-19.

**Table 1:** Reported feelings of isolation of older adult respondents in the year 2020, organized by demographic characteristics.

		**Hardly ever**	**Some of the time**	**Often**	**Chi-square *p*-value**
Age group	65–74 (*n* = 631)	34%	51%	15%	n.s
	75+ (*n* = 296)	37%	47%	17%	
Gender	Male (*n* = 449)	38%	48%	15%	n.s
	Female (*n* = 478)	32%	52%	16%	
Location	Rural (*n* = 144)	73%	21%	6%	n.s
	Urban (*n* = 783)	69%	27%	4%	
Housing	Live alone (*n* = 332)	34%	49%	18%	n.s
	Live with others (*n* = 572)	36%	51%	13%	
Relationship status	Not in partnership (*n* = 367)	36%	46%	17%	n.s
	In partnership (*n* = 560)	34%	52%	14%	
Income	Low (*n* = 229)	36%	46%_a_	18%_c_	0.021
	Middle (*n* = 422)	34%	50%_a, b_	16%_c_	
	High (*n* = 137)	34%	59%_b_	7%_d_	
Education	High school or less (*n* = 239)	36%	47%	16%	n.s
	Some post-secondary (*n* = 313)	33%	49%	18%	
	University graduate (*n* = 375)	36%	52%	13%	
BIPOC	No (*n* = 872)	35%	50%	15%	n.s
	Yes (*n* = 41)	32%	51%	17%	
Disability	No (*n* = 779)	35%	50%	14%_a_	n.s
	Yes (*n* = 142)	31%	47%	22%_b_	
Technology confidence	Confident (*n* = 669)	36%	50%	14%_a_	0.039
	Not confident (*n* = 172)	34%	44%	22%_b_	
Tech-savviness	Tech-savvy (*n* = 500)	37%_a_	49%	14%_a_	0.021
	Not Tech-savvy (*n* = 327)	30%_b_	50%	20%_b_	

*P-values for overall chi-squared relationship testing is reported (n.s, not significant). Each subscript letter indicates a significant difference (p < 0.05) in z-proportions testing with the demographic grouping (i.e., non-matching subscripts indicates significant proportional differences). Not all demographic groupings total 927 due to non-responses or preference to not answer*.

### Bivariate Relationship Effects

The sample was categorized into demographic groupings to compare feelings of isolation between various demographic characteristics (Table 1). Level of income was identified as having a significant relationship to feelings of isolation (*X*^2^ = 11.589, *df* = 4, *p* = 0.021). Specifically, the proportion of participants who reported feelings of isolation “Often” was significantly less for those who were identified as the highest income earners (6.6%), compared to those who were the middle (15.9%), and low-income earners (18.3%, *p* < 0.05). Furthermore, the proportion of those that reported feelings of isolation “Some of the time” was statistically greater in the high-income grouping (59.1%) compared to the low income (45.9%, *p* < 0.05).

Chi-squared testing for self-identified disability status and feelings of isolation returned non-significant overall, however, the proportion of those that reported feeling isolated “often” was greater for those who self-identified as a person living with a disability (21.8%) compared to those without (14.4%, *p* < 0.05).

Within all other demographic groupings, there were no significant relationships or differences in proportions in reported feelings of isolation. All demographic groups reported >60% of the respondents feeling isolated “Some of the time” or “Often” (range 62–68%).

When the 2,020 sample was sorted by participant's psychographic characteristics significant differences were evident. Individuals who reported that they were “not confident” in their use of currently available technologies were those who were reported feeling isolated “Often,” 22.1% compared to 14.2% of those who reported being “Confident” in their ability to use current technology (*X*^2^ = 6.473, *df* = 2, *p* = 0.039). Similarly, there was a significant relationship between reported feelings of isolation and individual's perception of their “Tech-savviness” (*X*^2^ = 7.698, *df* = 2, *p* = 0.021). Specifically, those who reported themselves as being “Not Tech-savvy” had a significantly greater proportion of participants report feeling isolated “Often” (20.2%) compared to 13.8% of those who reported themselves as “Tech-savvy” (*p* < 0.05). Those who reported being “Tech-savvy” also had a significantly greater proportion (37.2%) of those who reported feelings of isolation “Hardly ever,” compared to 30.3% of those who reported themselves as “Not Tech-savvy” (*p* < 0.05).

### Multinomial Multivariable Regression Effects

Findings for the unadjusted regression models are reported in Table 2. After adjustment for sociodemographic variables older adults who reported “hardly ever” feeling isolated were more likely to be those who identified as being a person without disability (OR 1.95, 95% CI 0.99–3.85) and those who reported themselves as tech-savvy (OR 2.04, 95% CI 1.10–3.77) when compared against those that felt isolated “often.” After adjustment, there were no significant differences demonstrated for those who reported feeling isolated “some of the time” compared to “often.”

**Table 2:** Adjusted and unadjusted multinomial logistic regression of feelings of isolation “Hardly ever” (*n* = 323) and “Some of the time” (*n* = 461) referenced to “Often” (*n* = 143).

	**Hardly ever**		**Some of the time**	
	**OR (95% CI)**			
	**Unadjusted**	**Adjusted**	**Unadjusted**	**Adjusted**
**Age group**				
65–74	1.02 (0.59, 1.76)	1.05 (0.61, 1.81)	1.01 (0.60, 1.71)	1.04 (0.62, 1.74)
75+	1.00	1.00	1.00	1.00
**Gender**				
Male	1.26 (0.75, 2.13)	1.24 (0.73, 2.08)	0.90 (0.55, 1.48)	0.89 (0.54, 1.46)
Female	1.00	1.00	1.00	1.00
**Location**				
Rural	1.28 (0.67, 2.45)	1.26 (0.66, 2.41)	1.02 (0.55, 1.93)	1.02 (0.55, 1.91)
Urban	1.00	1.00	1.00	1.00
**Housing**				
Live alone	0.68 (0.30, 1.51)	0.68 (0.31, 1.50)	0.82 (0.38, 1.77)	0.83 (0.39, 1.76)
Live with others	1.00	1.00	1.00	1.00
**Relationship status**				
Not in partnership	1.65 (0.72, 3.76)	1.76 (0.78, 3.98)	1.08 (0.49, 2.38)	1.13 (0.52, 2.46)
In partnership	1.00	1.00	1.00	1.00
**Income**				
Low	0.76 (0.29, 2.00)	0.76 (0.29, 1.99)	0.40 (0.16, 1.03)	0.40 (0.16, 1.01)
Middle	0.54 (0.23, 1.23)	0.54 (0.24, 1.22)	0.45 (0.21, 0.99)^*^	0.45 (0.21, 0.97)
High	1.00	1.00	1.00	1.00
**Education**				
High school or less	0.78 (0.40, 1.53)	0.79 (0.40, 1.54)	0.93 (0.50, 1.75)	0.94 (0.50, 1.77)
Some post-secondary	0.89 (0.49, 1.61)	0.86 (0.47, 1.55)	0.92 (0.52, 1.62)	0.90 (0.51, 1.58)
University graduate	1.00	1.00	1.00	1.00
**BIPOC**				
No	2.36 (0.64, 8.73)	2.24 (0.61, 8.26)	0.91 (0.31, 2.64)	0.89 (0.31, 2.58)
Yes	1.00	1.00	1.00	1.00
**Disability**				
No	2.05 (1.04, 4.06)^*^	1.95 (0.99, 3.85)^*^	1.39 (0.75, 2.57)	1.35 (0.73, 2.50)
Yes	1.00	1.00	1.00	1.00
**Technology confidence**				
Confident	0.93 (0.46, 1.86)	0.92 (0.46, 1.85)	1.29 (0.67, 2.47)	1.27 (0.67, 2.43)
Not confident	1.00	1.00	1.00	1.00
**Tech-savviness**				
Tech-savvy	2.07 (1.12, 3.84)^*^	2.04 (1.10, 3.77)^*^	1.35 (0.76, 2.38)	1.33 (0.75, 2.36)
Not Tech-savvy	1.00	1.00	1.00	1.00

* *p < 0.05*.

## Discussion

Our study aimed to investigate whether feelings of isolation were related to demographics characteristics that have previously been associated with greater risk of social isolation and loneliness. Additionally, we aimed to explore the effects of psychographic characteristics related to technology confidence on feelings of isolation. Our data set shows that many older adults are feeling isolated “Often” or “Some of the time” in 2020, regardless of various demographic factors that have been previously associated with increased isolation risk (6). We expected to see a disparity in reported levels of isolation based on demographic groupings that are associated with risk, with those belonging to at-risk demographic groupings having more reported frequency of feelings of isolation. Instead, we found that a large proportion of older adults are experiencing feelings of isolation at least “some of the time” regardless of almost all demographic factors evaluated.

Of the demographic factors evaluated in the bivariate analysis, income level, and self-reported disability status were the only demographic factors that indicated significantly different proportions of reported feelings of isolation. Where those who were low-income or middle-income earners, or had a disability reported feeling isolated “often” at a greater proportion than their demographic counterparts. These results align with our expectations and previous studies, where those with lower income and reported disability have been identified as those who are more likely to be socially isolated or report loneliness (6). Unexpectedly, however, our results also indicated that the high-income earners reported feeling isolated “some of the time” at a greater proportion than low-income earners. We speculate that this may be due to COVID-19 restriction and closures. With social and physical restrictions in place, these higher-income individuals may not have the opportunities for the interaction they may have previously expected or experienced. Despite this specific observation, the overall relationship of reported isolation and income level was found to have an inverse relationship, lower income was associated with more frequent feelings of isolation.

Our results for overall reported feelings of isolation are somewhat comparable to previous reports that evaluated feelings of isolation in older adults. Prior to COVID-19, a 2012 published study (9) identified that 18% of older adults reported feelings of isolation some of the time. Similarly, Hawley et al. (10) found that 18% of adults aged 62–91 reported frequent loneliness (with an additional 29% reporting occasional loneliness). Our data indicated that 15% reported feeling isolated “Often” and 50% “Some of the time.” While the proportion of individuals in our data set who reported feelings of isolation often is similar to previous studies, it is worth noting that the proportion of those who reported feelings of isolation some of the time is considerably greater than previous studies. This discrepancy may help explain why we did not observe as many significant differences in demographic factors as expected. It is likely, that because of the COVID-19 pandemic, individuals of many different characteristics who would not otherwise have reported feelings of isolation are now doing so (11). Meanwhile, those who were already feeling isolated before the pandemic are being “hidden” by the increased numbers who reported feeling isolated in the context of the pandemic. Essentially COVID-19 appears to have acted as a catalyst in reducing the demographic effect on feelings of isolation. However, we also explored the effects of two psychographic characteristics and found significant differences.

When evaluating the respondents by psychographic factors our results both within our bivariate and multivariate analysis indicated that those who felt more proficient in their abilities to use existing technologies were those who responded that they had less frequent feelings of isolation compared to those who felt less confident or tech-savvy. This finding is critical to consider in the context of COVID-19 and the transition to technology-based services and communication to protect public health and safety. As our results indicate, lower confidence in using current technology is related to feelings of isolation. Hence, supporting older adults to feel confident in using technology may be a significant factor in ameliorating experiences of social isolation during the COVID-19 pandemic. Previous studies have indicated that older adults can benefit from technology by reducing loneliness and increasing social contact (12–14), however, if individuals are not confident in using these technologies these benefits may not be fully realized. Rolandi et al. (15), support this theory through their findings; older adults who were trained in using social media platforms had less frequent feelings of loneliness and a more maintained level of social engagement during the pandemic lockdowns compared to untrained older adults.

Despite this previous evidence, we cannot be sure of the directionality of our observations. Do the older adults in our survey feel less isolated because they are using technology more during the pandemic to remain connected, and thus building a sense of mastery for technology and feeling less isolated, or does feeling less isolated emanate from the confidence that they have the capacity to remain connected through technology? Additionally, those that see themselves as socially isolated may have less access to technology and thus lower technology confidence. Furthermore, additional external factors related to COVID-19 not evaluated in our data could be influencing feelings of social isolation; technology confidence may not be the main driver of heightened frequency of feelings of social isolation.

It should also be mentioned that it is possible there may be unintended consequences to an increased use of technology for older adults. Research by Knowles and Hanson (16) demonstrates that some older adults do not find using technology rewarding in an of itself and consciously avoid “getting caught up in” digital life. Importantly, while some older adults may find features like social media to be useful for keeping in contact with family, many often feel social networking is time-wasting and have an aversion to being glued to one's mobile phone. Having to substitute normal face-to-face interactions with a digital stand in may lead some individuals to have negative association with these interactions and feel further isolated. That being said, in the context of a global pandemic where digital interaction may be the only safely available interaction, investigating how improving feelings of confidence and mastery for technology usage, in tandem with personal preferences, may be worthwhile to investigate in alleviating feelings of loneliness and social isolation.

While the research is based on a sample of Canadian older people, Canada is probably not untypical of many countries across the world. In all developed countries, physical distancing policies, and restrictions on social interaction were implemented, while many essential services were migrated to online platforms. As the COVID-19 pandemic continues to fluctuate, new variants emerge, along with the prospect of future pandemics, the longer-term implications of isolation, support for older adults in their use of technology and how these fit with personal preference and wants, should become a priority of policy and practice.

In conclusion, our results confirm that many older adults are experiencing feelings of isolation and contrary to previous studies, the majority of demographic factors examined within our study do not contribute to significant differences in feelings of isolation. Critically, in the context of a digital world during a pandemic, feelings of proficiency in using technology appears to be an important factor related to feelings of isolation. However, we note that social isolation among older adults may not so easily be cured by access and use of current technology. But, given that technology proficiency is a modifiable factor, and was significant after adjustment for demographic factors, future efforts to reduce social isolation could consider training programs for older adults to improve technology confidence.

## Data Availability Statement

The raw data supporting the conclusions of this article will be made available by the authors, without undue reservation.

## Ethics Statement

The studies involving human participants were reviewed and approved by data was provided to the authors for secondary analysis and approved by the Research Ethics Board at Simon Fraser University REB #30000195. Written informed consent for participation was not required for this study in accordance with the national legislation and the institutional requirements.

## Author Contributions

AS, DS, and AM conceived the study, and were part of overall direction and planning. BH analyzed the data with input from AS. BH wrote the manuscript with input from all authors.

## Funding

Financial support for this research was provided by AGE-WELL Network Centres of Excellence (www.agewell-nce.ca).

## Conflict of Interest

The authors declare that the research was conducted in the absence of any commercial or financial relationships that could be construed as a potential conflict of interest.

## Publisher's Note

All claims expressed in this article are solely those of the authors and do not necessarily represent those of their affiliated organizations, or those of the publisher, the editors and the reviewers. Any product that may be evaluated in this article, or claim that may be made by its manufacturer, is not guaranteed or endorsed by the publisher.
